# Evaluating the Utility of Smartphone-Based Sensor Assessments in Persons With Multiple Sclerosis in the Real-World Using an App (elevateMS): Observational, Prospective Pilot Digital Health Study

**DOI:** 10.2196/22108

**Published:** 2020-10-27

**Authors:** Abhishek Pratap, Daniel Grant, Ashok Vegesna, Meghasyam Tummalacherla, Stanley Cohan, Chinmay Deshpande, Lara Mangravite, Larsson Omberg

**Affiliations:** 1 Sage Bionetworks Seattle, WA United States; 2 Novartis Pharmaceuticals Corporation East Hanover, NJ United States; 3 Providence Multiple Sclerosis Center, Providence St Vincent Medical Center Portland, OR United States

**Keywords:** multiple sclerosis, digital health, real-world data, real-world evidence, remote monitoring, smartphone, mobile phone, neurodegeneration

## Abstract

**Background:**

Multiple sclerosis (MS) is a chronic neurodegenerative disease. Current monitoring practices predominantly rely on brief and infrequent assessments, which may not be representative of the real-world patient experience. Smartphone technology provides an opportunity to assess people’s daily-lived experience of MS on a frequent, regular basis outside of episodic clinical evaluations.

**Objective:**

The objectives of this study were to evaluate the feasibility and utility of capturing real-world MS-related health data remotely using a smartphone app, “elevateMS,” to investigate the associations between self-reported MS severity and sensor-based active functional tests measurements, and the impact of local weather conditions on disease burden.

**Methods:**

This was a 12-week, observational, digital health study involving 3 cohorts: self-referred participants who reported an MS diagnosis, clinic-referred participants with neurologist-confirmed MS, and participants without MS (controls). Participants downloaded the elevateMS app and completed baseline assessments, including self-reported physical ability (Patient-Determined Disease Steps [PDDS]), as well as longitudinal assessments of quality of life (Quality of Life in Neurological Disorders [Neuro-QoL] Cognitive, Upper Extremity, and Lower Extremity Function) and daily health (MS symptoms, triggers, health, mobility, pain). Participants also completed functional tests (finger-tapping, walk and balance, voice-based Digit Symbol Substitution Test [DSST], and finger-to-nose) as an independent assessment of MS-related cognition and motor activity. Local weather data were collected each time participants completed an active task. Associations between self-reported baseline/longitudinal assessments, functional tests, and weather were evaluated using linear (for cross-sectional data) and mixed-effects (for longitudinal data) regression models.

**Results:**

A total of 660 individuals enrolled in the study; 31 withdrew, 495 had MS (n=359 self-referred, n=136 clinic-referred), and 134 were controls. Participation was highest in clinic-referred versus self-referred participants (median retention: 25.5 vs 7.0 days). The top 5 most common MS symptoms, reported at least once by participants with MS, were fatigue (310/495, 62.6%), weakness (222/495, 44.8%), memory/attention issues (209/495, 42.2%), and difficulty walking (205/495, 41.4%), and the most common triggers were high ambient temperature (259/495, 52.3%), stress (250/495, 50.5%), and late bedtime (221/495, 44.6%). Baseline PDDS was significantly associated with functional test performance in participants with MS (mixed model–based estimate of most significant feature across functional tests [β]: finger-tapping: β=–43.64, *P*<.001; DSST: β=–5.47, *P*=.005; walk and balance: β=–.39, *P*=.001; finger-to-nose: β=.01, *P*=.01). Longitudinal Neuro-QoL scores were also significantly associated with functional tests (finger-tapping with Upper Extremity Function: β=.40, *P*<.001; walk and balance with Lower Extremity Function: β=–99.18, *P*=.02; DSST with Cognitive Function: β=1.60, *P*=.03). Finally, local temperature was significantly associated with participants’ test performance (finger-tapping: β=–.14, *P*<.001; DSST: β=–.06, *P*=.009; finger-to-nose: β=–53.88, *P*<.001).

**Conclusions:**

The elevateMS study app captured the real-world experience of MS, characterized some MS symptoms, and assessed the impact of environmental factors on symptom severity. Our study provides further evidence that supports smartphone app use to monitor MS with both active assessments and patient-reported measures of disease burden. App-based tracking may provide unique and timely real-world data for clinicians and patients, resulting in improved disease insights and management.

## Introduction

### Multiple Sclerosis

Multiple sclerosis (MS) is a chronic neurodegenerative disease that affects more than 2 million people worldwide, with prevalence rates equating to greater than 400,000 cases in the United States [1,2]. Symptoms of MS can affect motor function, sensation, cognition, and mood [3], and substantially impact quality of life (QoL) [4]. The combination of MS symptoms, their severity, and the course of disease varies between individual patients and can be affected by environmental factors, such as temperature, vitamin D stores, stress [1,5-10], and comorbidities, including depression, diabetes, cardiovascular disease, cancer, and autoimmune conditions [11,12]. Despite the heterogeneity of MS, certain symptoms and triggers are common among patients; for example, fatigue is reported as a symptom in approximately 75% of patients [13], and elevated temperature is estimated to cause transient symptom worsening in up to 80% [9].

### Patient Monitoring and Digital Tools

Routine clinical care for MS typically involves brief assessments performed during infrequent neurologist visits and, hence, often relies on retrospective self-reporting of symptoms and treatment responses, which can be subject to recall bias or affected by MS-associated cognitive impairment [14-17]. As such, this approach may fail to fully capture an individual’s day-to-day experience of living with MS and contribute to reduced accuracy and timeliness in detecting changes in symptom burden, disease severity/relapses, therapeutic outcomes, and the need for timely therapeutic agent change [17,18], Furthermore, while patient-reported outcomes (PROs) are increasingly used in MS clinical care, there is a lack of universal guidance on MS-specific PROs, and their usage and interpretation can differ between individual clinicians [17]. These challenges in monitoring MS highlight an unmet need for more effective, patient-centered tools that are able to capture the daily lived experience of disease outside of episodic clinic visits [19].

While a number of studies have used web-based tools to collect MS health data through patient diaries and electronic PROs [20,21], there is a growing need to tailor these digital health tools to the needs of patients with MS; this includes developing sensor-based assessments of MS disease severity and evaluating the impact of environmental factors, such as weather, stress, and sleep impairment on MS burden [22,23]. By bridging the gap between episodic clinical observation and the real-world experience of MS, these digital tools have the potential to improve self-reporting and disease monitoring, and provide a more comprehensive assessment of disease trajectories. In turn, this may support clinicians in making disease management recommendations to patients with MS, as well as other diseases that have heterogeneous and variable symptoms over time [24].

The ubiquity of smartphones with built-in sensors provides an opportunity to address the growing need for real-time disease monitoring. A number of previous studies have demonstrated the feasibility of smartphones in collecting health data in a real-time, real-world setting from patients across a range of disease areas including asthma, diabetes, depression, and Parkinson disease [25-30]. In addition to monitoring symptoms and triggers, these studies have identified geographic and environmental factors related to disease severity, and have been reported by patients to have a positive impact on their disease management [26,27,29]. Several digital health studies have already been undertaken in patients with MS with encouraging results; data suggest that smartphone technology can be effectively leveraged to monitor MS symptom severity, QoL, and medication usage, enabling patients to play an active role in disease management [23,31-35]. The recent FLOODLIGHT study has also shown that smartphone-based active testing can be used to remotely monitor motor function and capture MS symptoms, thus providing a more accurate assessment of MS in the real world [23,34]. However, a number of these studies have involved partially remote designs that include scheduled clinic visits at predetermined time points [32,34], which may limit widespread usage and participation. To this end, additional studies are required to build on these existing data and further assess digital health tools in a large, remote population of patients with MS.

### Objective

The main objective of this study was to evaluate the feasibility and utility of gathering MS-related health information from a large, remotely enrolled cohort using a dedicated smartphone app and to monitor study participants over a 12-week period. The study app, “elevateMS,” was developed through a user-centered design process. Secondary objectives were to examine the relationship between disease severity and QoL, measured via PROs and performance in sensor-based active functional tests, and to investigate the impact of local weather conditions on variations in MS symptoms and severity.

## Methods

### Study Design

This was a 12-week observational, prospective pilot digital health study using data collected from a dedicated smartphone app, elevateMS. The app was developed using a patient-centered design process with MS patient advisors (Multimedia Appendix 1) and was freely available to download from the Apple App Store. Enrollment spanned from August 2017 to October 2019, and participants were required to be aged 18 years or older, reside in the United States, and use an iPhone 5 or newer device.

### Study Participants

**Figure 1:**
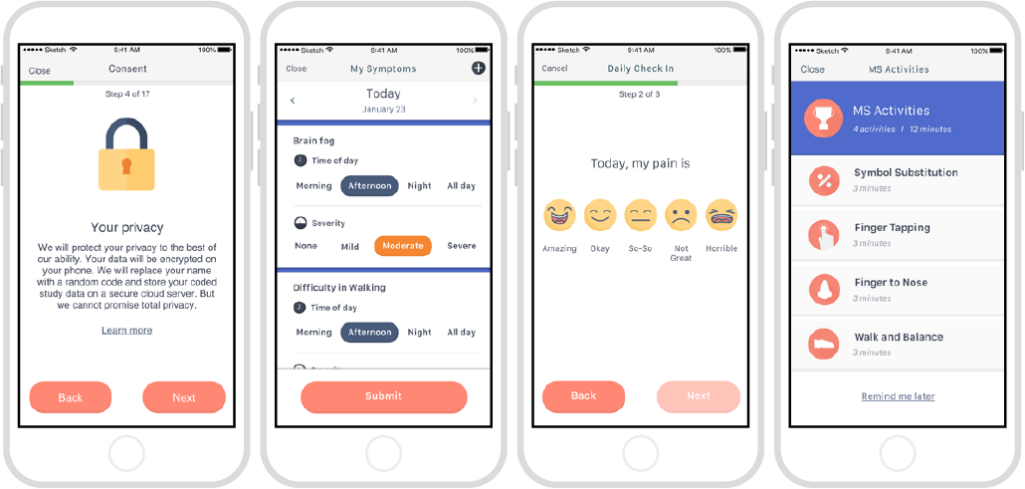
Example screenshots from the elevateMS study app.

Participants were openly recruited through word of mouth, press releases, online advertisements, and the study website [36] and grouped into 2 cohorts: individuals without MS (controls) and individuals who self-reported diagnosis of MS (self-referred). A third, “clinic-referred” cohort, with a neurologist-confirmed MS diagnosis, was also recruited through information flyers distributed at 3 MS treatment centers. Ethical approval was granted by the Western Institutional Review Board, and enrollment, informed consent, and data collection were carried out electronically through the study app (Figure 1) [37].

Participants had to make an active choice to complete the consent process, and no default option was presented. Participants were also given the option to share their data only with the elevateMS study team and partners (share narrowly), or more broadly with qualified researchers worldwide [38].

### Data Collection

elevateMS primarily targeted collection of real-world data from participants with MS. This included self-reported measures of symptoms and health via optional “check-in” surveys, and independent assessments of motor function via sensor-based active functional tests. Participants were encouraged to complete check-in surveys on a daily basis and were notified to perform more comprehensive functional tests once a week. Local weather data were collected every time an assessment was performed. The data collected through elevateMS and the frequency at which each element was recorded are summarized in Table 1. With the exception of overall physical ability, which was a baseline-only assessment, all data were collected longitudinally at various intervals over the 12-week study duration.

**Table 1:** Data collected through the elevateMS study app.

Data source		Timeline of data collection	
**Baseline demographics**			
	Sociodemographic data (age, gender, race, education, health insurance, employment status, geographic location)	Day 2	
	MS^a^ disease characteristics (diagnosis, medication, family history)	Day 1	
**Patient-reported outcomes**			
	Overall physical ability^b^	Day 2	
	Check-in survey: MS symptoms and triggers	Daily	
	Check-in survey: health, mobility, pain^c^	Daily	
	Short-form Neuro-QoL^d^ domains	Every third functional test targeting that domain (Cognitive Function domain: DSST; Upper Extremity Function domain: finger-tapping/finger-to-nose; Lower Extremity Function domain: walk and balance)	
**Active functional test**			
	Finger-tapping	Weekly^f^	
	Walk and balance	Weekly^f^	
	DSST^e^	Weekly^f^	
	Finger-to-nose	Weekly^f^	
**Local weather data**			
	Temperature	Every time a test was performed	
	Humidity	Every time a test was performed	
	Cloud coverage	Every time a test was performed	
	Atmospheric pressure	Every time a test was performed	

^a^MS: multiple sclerosis.

^b^Based on a truncated 4-point Patient-Determined Disease Steps scale, administered at baseline to all patients with MS (Normal, Mild Disability, Moderate Disability, Gait Disability).

^c^Based on a 5-point Likert scale (Health: Amazing, Okay, So-So, Not great, Horrible; Mobility: Excellent, Very good, Good, Not great, Horrible; Pain: None, Mild, Moderate, Severe, Horrible).

^d^Neuro-QoL: Quality of Life in Neurological Disorders.

^e^DSST: Digit Symbol Substitution Test.

^f^With option for participants to complete more frequently.

Participants with MS completed optional daily check-in surveys to record their individual symptoms and triggers, and to assess their health, mobility, and pain using a 5-point Likert scale. Self-reported disease severity was determined using a truncated 4-point Patient-Determined Disease Steps (PDDS) scale to assess overall physical ability (Normal, Mild Disability, Moderate Disability, Gait Disability) [39]. The impact of MS on daily function was also assessed using 3 short-form domains of the previously validated Quality of Life in Neurological Disorders measurement tool (Neuro-QoL; Cognitive Function, Upper Extremity Function, and Lower Extremity Function; Multimedia Appendix 2) [40-42]. Raw Neuro-QoL scores were converted to standardized Theta (T) scores using standard scoring protocols [42], and subsequently classified into discrete, clinically relevant categories (Normal, Mild, Moderate, and Severe) based on past research [43-45]. All participants carried out active functional tests, which used smartphone sensors as a proxy for traditional symptom measurements. These tests included:

1. The finger-tapping test, where participants repeatedly tapped between 2 circles with alternating fingers as fast as possible for 20 seconds, to measure dexterity, speed, and abnormality in movement.
2. The walk and balance test, where participants walked for 20 seconds with their iPhone in their pocket, then stood still for 10 seconds, to assess gait, posture, stability, and balance;
3. The voice-controlled Digit Symbol Substitution Test (DSST) [46], where participants used the microphone to record answers to measure cognitive function.
4. The finger-to-nose test, where participants extended their arm while holding their iPhone and then touched the phone screen to their nose repeatedly, to measure kinetic tremor and dysmetria in each hand.

Raw data collected from sensor-based active functional tests were transformed using the mhealthtools package, an open-source feature engineering pipeline [47]. This process generated features for each functional test related to different aspects of a participant’s health state; for example, the finger-tapping test comprised over 40 features, including number of taps, frequency, and location drift. See Multimedia Appendix 3 for specific examples and the elevateMS Feature Definitions webpage for a full list of features [48]. In addition to extracting features, the mhealthtools pipeline also filtered out records lacking data [47].

The data contributed by participants, as well as scheduling of in-app active functional tests, were managed using an open-source platform developed and maintained by Sage Bionetworks [49].

### Statistical Analyses

Descriptive statistics were used to summarize and compare the baseline demographics and MS disease–related characteristics in the study cohort.

User-engagement data were collected and analyzed to understand participant demographics, retention, and compliance in the study. Retention analysis was performed under the definition that participants were considered active in the study if they completed at least one survey or sensor-based test in a given week. The total duration in the study was determined by the number of days between the first and last test. Participants’ weekly compliance was assessed using a more stringent cut off than weekly retention; a participant was considered minimally compliant if he/she completed at least one out of four weekly sensor-based active functional tests. Overall retention (ie, total duration a participant remained in the study) was examined across the 3 cohorts (controls, self-referred participants with MS, and clinic-referred participants with MS) using Kaplan–Meier plots. A log-rank test was used to compare the retention difference between the 3 cohorts. The impact of baseline demographics and MS disease characteristics on participant retention was assessed using a Cox proportional hazards model. Each covariate of interest was tested independently, including an interaction term for the clinical referral status. The assumption of the proportional hazard model was tested using scaled Schoenfeld residuals. Finally, per the study protocol, participants were expected to remain in the study for 12 weeks, and thus all user-engagement analysis was limited to the first 12 weeks of study participation.

Linear regression models were used to test for association between participants’ self-reported demographics, baseline physical ability (collected once during the onboarding), and the median value of all features generated for each of the 4 sensor-based active functional tests. To test for association between longitudinal PRO assessments (ie, Neuro-QoL results and daily check-ins) and active functional test performance, as well as the potential impact of local weather conditions, a linear mixed-effects (LME) modeling approach was used to account for the subject-level heterogeneity. Prior to modeling, the PRO data were aligned with more frequently administered functional tests by aggregating the average value for all features per week. LME models were fit using the R package lme4, version 1.1-23 [50] with combinations of fixed and random effects. Because of a significant amount of missing responses in sociodemographic information for participants (see Multimedia Appendix 4), some LME models did not converge, and therefore a simpler LME model accounting for participant-level random effect only was used. Statistical significance (*P*-values) for LME models was determined using the Satterthwaite degrees of freedom method through the lmerTest package (version 3.1-1) [51]. For analysis conducted using LME models, we report estimates (β) of all fixed effects covariates along with *P*-values. In addition, *P*-values from ANOVA test conducted to assess significant differences in LME model fixed effect estimates (regardless of the individual factor levels) are also reported. In case a functional test was due to be completed by both hands, the LME models also accounted for variations due to left- and right-hand differences. As participants with MS completed active functional tests at different frequencies, participation rates, sample size, and number of data points varied between analyses. We also conducted sensitivity analyses to evaluate the impact of extreme Neuro-QoL and health, mobility, and pain categories on association results; this involved excluding functional test scores that mapped to the Severe Neuro-QoL category and excluding health, mobility, and pain scores that mapped to the Horrible category. All *P*-values were corrected for multiple testing and false positives using the Benjamini–Hochberg procedure. All analyses were performed using open-source statistical analysis framework R (version 3.5.2; R Foundation for Statistical Computing) [52].

### Data Availability

Complete results from this analysis are available online through the accompanying elevateMS study portal [53]. Additionally, individual user-level raw data for those participants who consented to share their data broadly with qualified researchers worldwide is also available under controlled access through the study portal [54].

## Results

### Study Population

**Figure 2:**
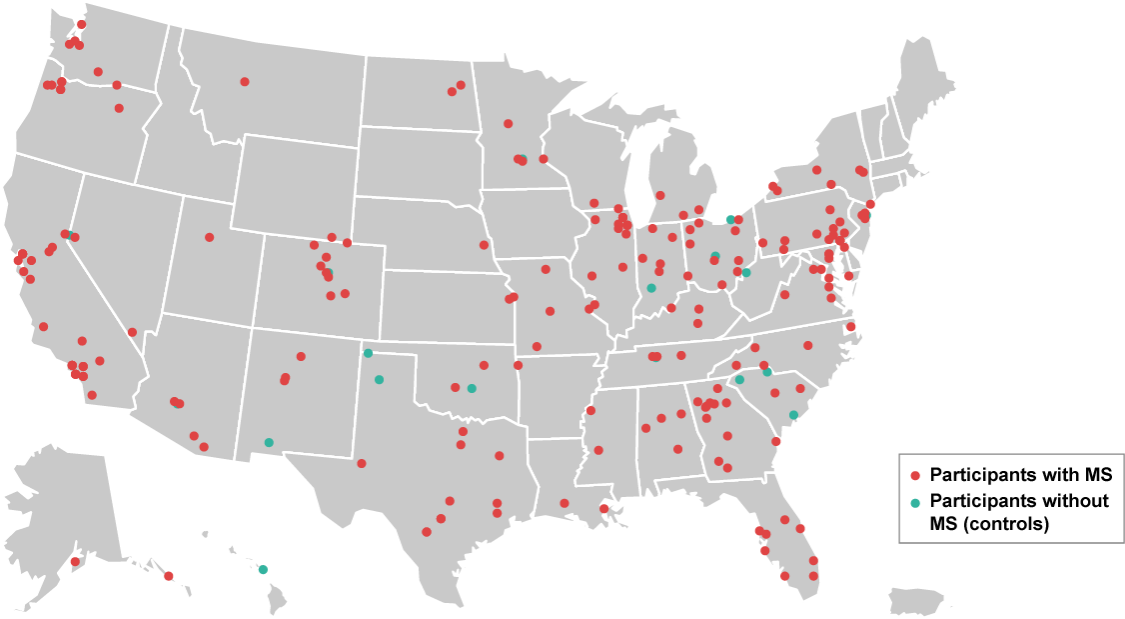
Geographic locations of participants. Dots (n=329) represent the location of those participants who continued in the study beyond initial enrollment and provided the first three digits of their zip code during the collection of demographic information on Day 2. One dot is included for each location, with participants with the same first three digits of the zip code shown under the same dot.

The elevateMS app was released in August 2017 through the Apple App Store and enrolled participants on a rolling basis until October 2019. A total of 660 participants enrolled in the study, of which 31 selected to withdraw with no reason provided. Of the remaining 629 participants, 134 (21.3%) were controls (self-reported as not having MS) and 495 (78.7%) were participants with MS. Of the 495 participants with MS, 359 (72.5%) self-referred to the study with a self-reported MS diagnosis and 136 (27.5%) were referred from 3 clinical sites and had a neurologist-confirmed MS diagnosis. Participants were located across the United States (Figure 2), with a mean (SD) age of 39.34 (11.41), 45.20 (11.64), and 48.93 (11.20) years in the control, self-referred, and clinic-referred cohorts, respectively. A summary of baseline sociodemographic data is presented in Table 2. Further information on missing responses in demographic data is presented in Multimedia Appendix 4.

Baseline disease characteristics for the self-referred and clinic-referred participants with MS are shown in Table 3. Most participants reported relapsing–remitting MS; this included 83.6% (300/359) of the self-referred cohort and 90.4% (123/136) of the clinic-referred cohort. Infusion disease-modifying therapy was the most common treatment received by both self-referred (116/359, 32.3%) and clinic-referred (64/136, 47.1%) participants.

**Table 2:** Baseline sociodemographic characteristics of study participants.^a^

Characteristic		Controls (N=134)	Participants with MS^b^ (self-referred; N=359)	Participants with MS (clinic-referred; N=136)
Age (years), mean (SD)		39.34 (11.41)	45.20 (11.64)	48.93 (11.20)
**Gender**				
	Female	27 (64.3)	154 (73.3)	78 (84.8)
	Male	15 (35.7)	56 (26.7)	14 (15.2)
**Race**				
	Asian	6 (13.6)	4 (1.9)	0 (0.0)
	Black African	1 (2.3)	13 (6.1)	9 (9.8)
	Caucasian	26 (59.1)	182 (85.4)	74 (80.4)
	Latino Hispanic	5 (11.4)	9 (4.2)	5 (5.4)
	Other	6 (13.6)	5 (2.3)	4 (4.3)
**Education**				
	College degree	16 (37.2)	123 (57.5)	55 (60.4)
	High-school diploma/GED^c^	4 (9.3)	16 (7.5)	7 (7.7)
	Postgraduate degree	23 (53.5)	70 (32.7)	29 (31.9)
	Other	0 (0.0)	5 (2.3)	0 (0.0)
**Health insurance**				
	Government insurance	3 (7.0)	65 (30.5)	19 (20.7)
	Employer insurance	30 (69.8)	100 (46.9)	55 (59.8)
	No insurance	1 (2.3)	2 (0.9)	2 (2.2)
	Other	9 (20.9)	46 (21.6)	16 (17.4)
**Employment status**				
	Full-time	30 (69.8)	93 (43.5)	41 (44.6)
	Part-time	3 (7.0)	17 (7.9)	11 (12.0)
	Retired	3 (7.0)	18 (8.4)	10 (10.9)
	Disabled	4 (9.3)	62 (29.0)	19 (20.7)
	Unemployed	0 (0.0)	10 (4.7)	3 (3.3)
	Other	3 (7.0)	14 (6.5)	8 (8.7)

^a^All data shown are n (%), unless otherwise stated. Percentages were calculated based on the total number of participants who provided a response and excluded missing information. See Multimedia Appendix 4 for further details on missing results, including the number and proportion of participants who did not provide responses.

^b^MS: multiple sclerosis.

^c^GED: General Educational Development.

**Table 3:** Baseline disease characteristics of study participants with MS.

Characteristic^a^		Participants with MS (self-referred; N=359)	Participants with MS (clinic referred; N=136)	
**MS^b^ diagnosis**				
	Relapsing–remitting	300 (83.6)	123 (90.4)	
	Primary progressive	34 (9.5)	6 (4.4)	
	Secondary progressive	25 (7.0)	5 (3.7)	
	Not sure	0 (0.0)	2 (1.5)	
**Current DMT^c^**				
	Infusion	116 (32.3)	64 (47.1)	
	Injection	83 (23.1)	24 (17.6)	
	Oral	114 (31.8)	40 (29.4)	
	None	46 (12.8)	6 (4.4)	
	Missing	0 (0.0)	2 (1.5)	
**MS family history**				
	Yes	77 (21.4)	23 (16.9)	
	No	251 (69.9)	104 (76.5)	
	Not sure	31 (8.6)	9 (6.6)	
**Overall physical ability^d^**				
	Normal	101 (28.1)	52 (38.2)	
	Gait disability	85 (23.7)	24 (17.6)	
	Mild disability	104 (29.0)	38 (27.9)	
	Moderate disability	69 (19.2)	20 (14.7)	
	Missing	0 (0.0)	2 (1.5)	
**Duration of disease**				
	Years since diagnosis, mean (SD)	11.14 (8.86)	14.29 (8.89)	
**Duration of treatment**				
	Years since first DMT, mean (SD)	10.09 (7.97)	13.07 (7.92)	

^a^All data shown are n (%), unless otherwise stated.

^b^MS: multiple sclerosis.

^c^DMT: disease-modifying therapy.

^d^Based on truncated 4-point Patient Determined Disease Steps scale.

### Participant Engagement

**Figure 3:**
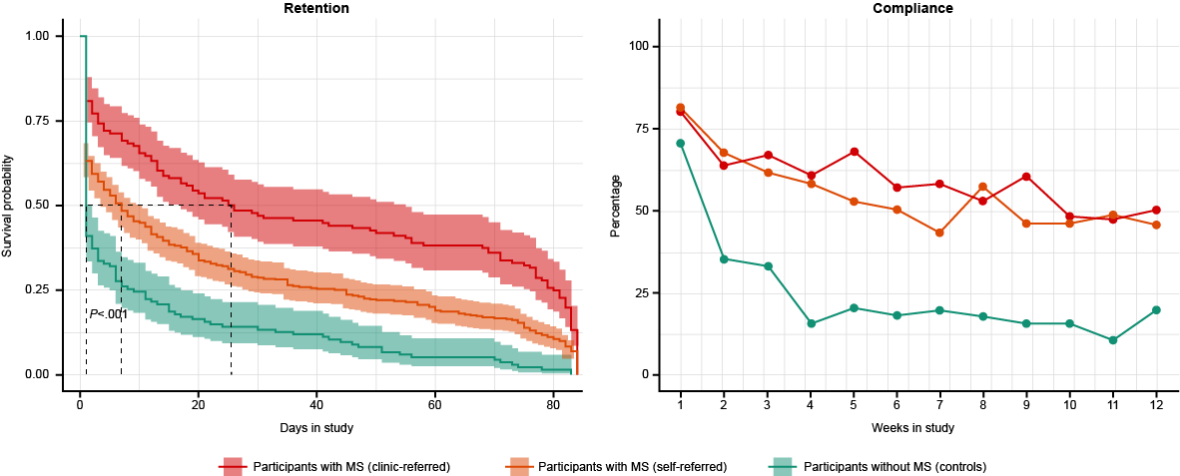
elevateMS user engagement. Participant retention (median number of days in the study) and compliance (completion of at least one out of four sensor-based active functional tests per week) across the three study cohorts. MS: multiple sclerosis.

Median study retention was significantly higher in clinic-referred participants with MS (25.5 days [95% CI 17.0-55.0]) compared with self-referred participants with MS (7.0 days [95% CI 4.0-11.0]) and controls (1.0 day [95% CI 1.0-2.0 days]; *P*<.001; Figure 3). Compliance, defined in this study as the completion of at least one sensor-based active functional test per week, decreased over time in all cohorts; from Week 1 to Week 12, compliance fell from 80.2% to 50.0% for the clinic-referred cohort, from 81.1% at Week 1 to 46.1% for the self-referred cohort, and from 70.9% to 20.0% in the control cohort (Figure 3). Given the lack of ongoing engagement in the control group and the fact that elevateMS primarily targeted participants with MS, the control cohort was not included in subsequent data analyses, and results from self-referred and clinic-referred participants with MS were pooled for analysis.

For patients with MS, participation in all tasks (both sensor-based active functional tests and daily check-in surveys) was highest in Weeks 1 and 2, then decreased over time (Multimedia Appendix 5). When using the elevateMS app, participants with MS completed the active functional tests (median 40% of overall individual activity; IQR 30.3) the most, followed by reporting MS symptoms and triggers (median 33.3% of overall individual activity; IQR 33) and completing daily check-in surveys (median 22.3% of overall individual activity; IQR 18.2; Multimedia Appendix 6). The most common self-reported symptoms were fatigue, weakness, memory/attention issues, and difficulty walking, and the most common self-reported triggers were high ambient temperature, emotional stress, and going to bed late, all of which were experienced on at least one occasion by more than half of participants with MS (Multimedia Appendix 7). Data collected from sensor-based active tests were transformed into features and filtered for validity using the mhealthtools pipeline [47] (see Multimedia Appendix 8 for further details).

### Relationship Between Baseline Physical Ability and Performance in Active Functional Tests

**Figure 4:**
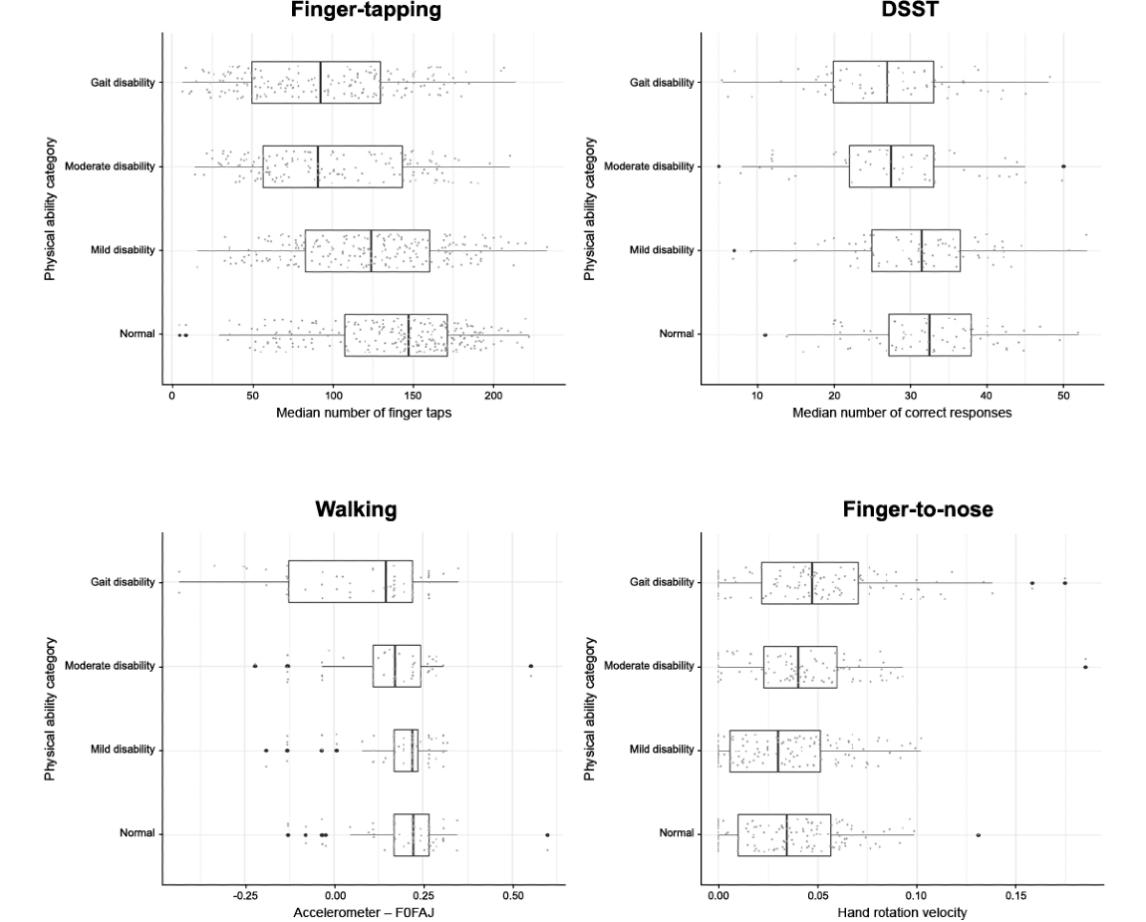
Association between baseline characteristics and functional test performance in participants with MS. DSST (Digit Symbol Substitution Test): decrease in number of correct DSST responses with increased baseline physical disability; F0FAJ: frequency at which the maximum peak of the Lomb-Scargle periodogram occurred for the average acceleration series, with frequencies limited to 0.2-5 Hz; Finger-tapping: decrease in median number of finger taps with increased baseline physical disability; Finger-to-nose: increase in hand rotation velocity tremor feature with increased baseline physical disability; MS: multiple sclerosis; PDDS: Patient-Determined Disease Steps; Walking: decrease in F0FAJ accelerometer results with increased baseline physical disability.

Higher physical disability at baseline was associated with significantly worse performance (*P*<.001; top associations listed below) in multiple sensor-based active functional tests. For each active functional test, a subset of the most significantly associated features is presented here (Figure 4 and Multimedia Appendix 9); the full list of per-feature results is available via Synapse, an online data repository managed by Sage Bionetworks [55]. For the finger-tapping assay, a reduced number of finger taps was statistically significantly associated with baseline physical ability category (β_gait disability vs normal_=–43.64, *P*<.001). Similarly, fewer correct responses in the voice-based DSST task was significantly associated with baseline physical ability (β_gait disability vs normal_=–5.47, *P*=.005). Worse performance in the walking test, based on a feature derived from the device accelerometer (F0FAJ) [48], was statistically significantly associated with low baseline physical ability (β_gait disability vs normal_=–0.39, *P*=.001). For the finger-to-nose test, a tremor feature derived from capturing the hand rotation velocity was also found to be significantly associated with baseline physical ability (β_gait disability vs normal_=0.01, *P*=.01). Balance features were not significantly associated with baseline physical ability (data not shown). Notably, the feature most associated with performance from the finger-tapping assay (median number of finger taps) was also significantly associated with several sociodemographic characteristics in participants with MS, including age group (*P*<.001), education (*P*=.001), duration of treatment (*P*=.004), and duration of disease (*P*=.009; Multimedia Appendix 9). Additionally, baseline physical ability was significantly associated with duration of treatment (*P*=.003), duration of disease, employment status, type of health insurance, and age group (all *P*<.001) in participants with MS (Multimedia Appendix 10).

### Relationship Between Neuro-QoL Domains and Performance in Active Functional Tests

**Figure 5:**
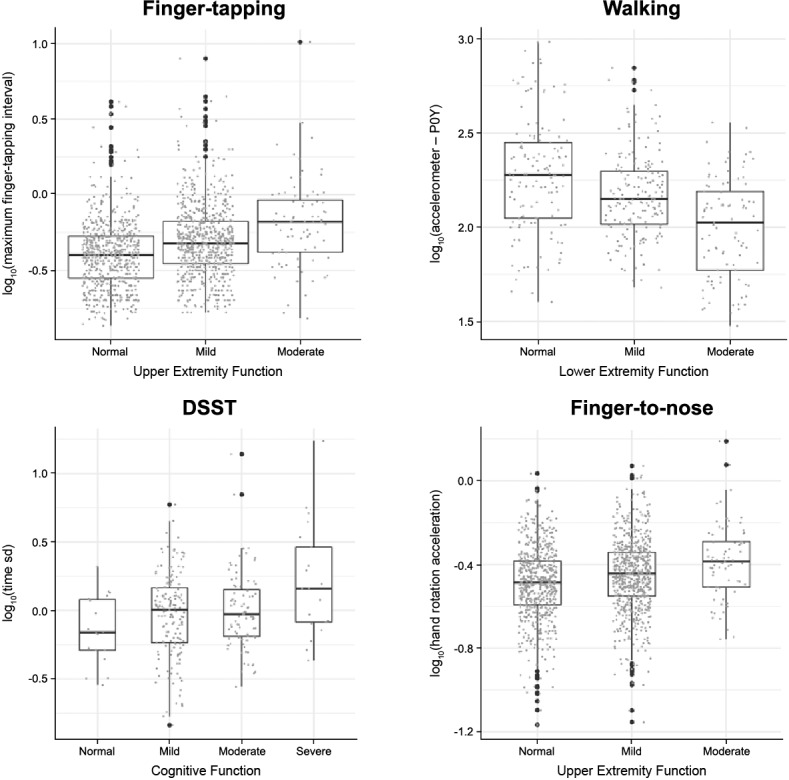
Association between Neuro-QoL^TM^ domains and functional test performance in participants with MS. Neuro-QoL categories comprising <5% of total participants were not plotted. DSST (Digit Symbol Substitution Test): increase in DSST response time with increased severity in Neuro-QoL Cognition domain. Finger-tapping: increase in maximum finger tapping interval with increased severity in the Neuro-QoL Upper Extremity Function domain; Finger-to-nose: increase in hand rotation acceleration tremor feature with increased severity in Neuro-QoL Upper Extremity Function domain; MS: multiple sclerosis; Neuro-QoL: Quality of Life in Neurological Disorders; P0Y: maximum power in the inspected frequency interval of the Lomb–Scargle periodogram for the Y acceleration series (0.2–5 Hz); Walking: decrease in accelerometer-derived feature (P0Y) with increased severity in Neuro-QoL Lower Extremity Function domain.

Performance in sensor-based active functional tests was also significantly associated (*P*<.001; top associations listed below) with the 3 short-form Neuro-QoL surveys (Cognitive Function, Upper Extremity Function, and Lower Extremity Function) that were administered to participants at several points during the course of the study. For each test, a subset of the top most significantly associated features are presented here (Figure 5 and Multimedia Appendix 11), with the full list of per-feature comparisons available online via Synapse data repository [56]. Performance in the finger-tapping test was significantly associated with Upper Extremity Function domain scores (β_moderate vs normal_=0.40 seconds, *P*<.001) of the Neuro-QoL. For the walking test, a feature derived from the device accelerometer (P0Y) [48] was found to be significantly associated with the Neuro-QoL Lower Extremity Function domain scores (β_moderate vs normal_=–99.18 seconds, *P*=.02). Increased severity in the Neuro-QoL Cognitive and Lower Extremity Function domains was also significantly associated with the variation in time taken to complete the voice-based DSST task (Cognitive Function: β_severe vs normal_=1.60 seconds, *P*=.03; Lower Extremity Function: β_severe vs normal_=10.31 seconds, *P*<.001). Finally, for the finger-to-nose test, a feature derived from capturing the hand rotation acceleration using the device gyroscope was significantly associated with the Neuro-QoL Upper Extremity Function domain (β_moderate vs normal_=.11, *P*=.003). Additional sensitivity analyses were performed to assess the impact of Neuro-QoL scores that mapped to the Severe Neuro-QoL category. While excluding Neuro-QoL scores in severe category did not change the main findings for the finger-tapping, walk and balance, and finger-to-nose tests, the analysis showed that the significant association of Cognitive Function and Lower Extremity Function with DSST was mainly driven by those participants reporting severe Neuro-QoL outcomes (Multimedia Appendix 11).

### Impact of Local Weather Conditions on Performance in Active Functional Tests and Daily Check-In PROs

**Figure 6:**
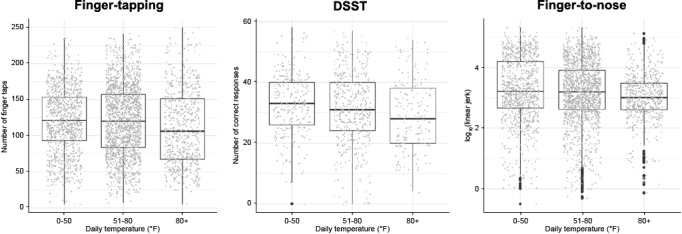
Association between daily temperature and functional test performance in participants with MS. Finger-tapping: decrease in number of finger taps with increased temperature. DSST (Digit Symbol Substitution Test): decrease in number of correct DSST responses with increased temperature. Finger-to-nose: decrease in accelerometer-derived linear jerk tremor feature derived with increased temperature. MS: multiple sclerosis.

Local weather data were captured each time a participant completed a functional test and were found to be significantly associated (*P*<.001; top associations listed below) with respective test performance (Figure 6 and Multimedia Appendix 12) [57]. The local temperature elevations at the time of test completion negatively impacted participant’s performance to the greatest extent, with a significant difference in performance in the finger-tapping test observed (β=–.14, *P*<.001). For example, with a 30°F increase in temperature (ie, from 50°F to 80°F), the participant’s performance dropped by an average of 4.3 finger-taps (β × 30°F). Similarly, performance in the voice-based DSST and finger-to-nose tests was significantly associated with increased temperature (β=–.06, *P*=.009 and β=–53.88, *P*<.001, respectively). In contrast to active functional test features, PROs recorded from daily check-in surveys were only moderately associated with local weather conditions (Multimedia Appendix 13) [58]. Sensitivity analysis further showed that the association between PROs and weather features was mainly driven by a small proportion of participants reporting extreme outcomes on the Likert scale (Multimedia Appendix 14) [59].

## Discussion

### Principal Results

The results from this observational, remote data collection study demonstrate the feasibility and utility of a decentralized method to gather real-world data about participant’s real-time life experience of MS through a digital health app, elevateMS. Compared with previous digital health studies in MS, elevateMS enrolled one of the largest remote cohorts with a self-reported or neurologist-confirmed diagnosis of MS from across the United States [23,31,32,34,35]. The sociodemographic characteristics of the enrolled patient cohort were broadly similar to the wider population of patients with MS [60,61]. Participation in elevateMS was also generally consistent with that of digital health studies in other disease areas [62]. However, to our knowledge, elevateMS is one of the first remote, digital studies to show the significant impact of clinical referral on overall engagement in a remote population [22]; clinic-referred participants with MS remained active in our study almost 3 times longer than self-referred participants with MS. Importantly, the digitally measured functional activity correlated with clinical outcomes and QoL.

Through the longitudinal collection of PROs, active functional test results, and local weather data, the elevateMS study demonstrates the importance of frequent, real-world assessments of MS disease manifestations outside of episodic clinical evaluations. Tracking of self-reported data identified the most common disease symptoms and triggers in patients with MS, as well as significant associations (*P*<.001) between performance in active functional tests and disease severity, measured by both PDDS and Neuro-QoL subdomain scores. Although PROs failed to capture the impact of local weather conditions, participants’ performance in various active functional test features was found to be significantly associated (*P*<.001) with local temperature patterns, with the worst performance observed at temperatures over 80°F. This supports the well-established link between increased temperature and MS [7-9] and demonstrates the sensitivity of these sensor-based tests in patient monitoring. Together, these results show the potential utility of active functional tests in capturing measurements of MS-related motor activity and assessing the impact of local environmental factors on disease symptoms and severity in a real-world setting.

A major strength of the elevateMS study is that it collected self-reported and self-administered measurements of MS health data from patients remotely. PROs are increasingly recognized as valid and meaningful clinical measures for disease monitoring and patient care across a range of therapeutic areas [63-72]. Within the MS field, improved self-assessment and QoL reporting, particularly using electronic/digital tools such as elevateMS in a remote, unsupervised setting, has the potential to benefit both patients and clinicians by enhancing communication and understanding of individual patient needs, thus improving the overall patient experience and informing therapy selection [17,24,73,74]. This is particularly important given that a low level of concordance has been observed between patients and neurologists in recognizing MS relapses, assessing health status, and identifying QoL parameters that are of greatest concern to the individual patient [17,24,75].

Another strength of this study is that both PROs and active measurements were collected contemporaneously in a real-time and real-world setting, with a frequency far exceeding that obtainable in routine MS clinical care. This differentiates elevateMS from the episodic and retrospective periods of data collection that are characteristic of traditional, clinic-based care and often subject to recall bias. By capturing a more comprehensive body of data, such as the frequency of triggers, variability of symptoms, and effect of environmental conditions, elevateMS could enable the interaction between daily life stressors and MS severity to be better evaluated. Furthermore, by leveraging frequent and low-burden assessments, elevateMS and other digital health tools may facilitate regular patient monitoring between clinic visits. This could complement the existing clinical practice, by helping patients to record their symptoms, relapses, and medication usage more accurately and have an active role in their disease management; in turn, this has the potential to provide a more thorough assessment of disease, improve communication with health care providers, and ultimately support clinicians in developing personalized treatment plans [19,27,29,32,34,35]. More broadly and universally applied, this technology may also provide novel insights into the course of chronic and progressive conditions, as demonstrated by previous digital health studies in MS, asthma, and Parkinson disease [25-27,34]. Finally, as shown by elevateMS, digital health tools can be utilized to gather data from large, remote populations and could, therefore, offer unique opportunities to track and evaluate drug efficacy in a continuous, real-world setting through decentralized clinical trials [34].

### Limitations

Some potential limitations need to be considered when interpreting the results from this study. Given that participation required a specific smartphone, the study population may be subject to selection bias [62]; however, the sociodemographic characteristics available from the enrolled patient cohort are broadly representative of the general population with MS [60,61]. As this study largely focused on patients with MS, participation by the control cohort was low, and these data were not included in our analyses. Approximately two-thirds of the total MS cohort self-reported their disease status, and these data may be vulnerable to inaccuracies. However, prior studies have shown that self-reported MS diagnoses in the Pacific Northwest MS Registry were subsequently confirmed by neurology health care providers in more than 98% of cases [76]. In a second study, levels of disability were accurately self-reported by patients and comparable to neurologist ratings in 73% of cases [77]. Within the elevateMS study, data from both self-referred and clinic-referred patients with MS were pooled for analysis, thus precluding between-group comparisons. Future studies could evaluate these 2 patient cohorts separately to enable results to be analyzed in relation to clinical referral status.

We also observed a large degree of missing baseline demographic data within this pilot study, which may reflect the fact that this information was not collected until Day 2 of participation, in an attempt to reduce initial onboarding participant burden. This missing data could impact statistical inference, in particular the robustness of the LME models, as the random effect of many sociodemographic characteristics could not be accounted for. Given that a significant proportion of participants in remote app-based studies drop out during the first week, with the majority leaving on Day 1-2 [62], future studies should prioritize immediate collection of baseline demographic data and make this a compulsory step upon enrollment. This would ensure that demographic information can be fully evaluated in relation to app study results.

Given the limited, 12-week study participation period, we were not able to assess longer-term variations in MS symptoms or severity in this pilot study. Furthermore, this short window of observation meant that disease severity could be assessed in relation only to daily, and not seasonal, fluctuations. Going forward, longer study durations would provide greater opportunities for longitudinal disease monitoring and assessment of the impact of external lifestyle and environmental factors. This is particularly important in chronic diseases such as MS, where there is a risk of progression and unpredictable variability of symptoms or relapses over time [5].

The sample size and engagement of the elevateMS patient cohort were generally consistent with or higher than those in previously published remote digital studies undertaken across different therapy areas [28-30,62]. However, the overall participation in elevateMS was still low compared with digital studies in MS that included an in-person clinic visit [31,32]. The greatest proportion of activity within the elevateMS study app was spent on sensor-based active functional tests, followed by symptom and trigger surveys, and check-in surveys. Participation in specific app activities may have been affected by the time, effort, or frequency associated with that activity; for example, each of the 4 sensor-based active tests were administered on a weekly basis and could be completed in less than 1 minute, whereas each survey was administered daily, potentially making them more burdensome despite their short length. Future studies could further align the study protocol with participant needs and busy schedules employing user-centered co-design techniques. Review of compliance patterns also demonstrated that patients referred from and currently under the care of a provider were the most adherent participants, and future studies should focus on building studies around this cohort. Doing so will not only increase the likelihood of patient retention, but will also provide physician verification of the validity of responses. Additional studies are necessary to develop strategies, including the use of incentives to increase involvement and adherence of people who self-identify and self-refer, to expand the number of patient participants with greater assurance of long-term adherence.

Within the elevateMS study, clinic-referred participants demonstrated greater retention and compliance than the self-referred cohort, which suggests that participants are more likely to engage if they are encouraged by a clinician or aware of how their data may be used to inform and personalize their care. Based on these results, future digital health studies should be incorporated, as exploratory outcomes initially, within clinical trials to assess the applicability and utility of digital monitoring. Finally, it is known that comorbidities, as well as concurrent medication use, have a significant impact on MS patients [11,12,78,79]; details regarding comorbidities were not collected in this pilot study, but should be included in future long-term assessments.

### Comparison With Prior Work

In line with previous digital health feasibility studies, our results demonstrate that smartphone technology can be used to collect both sensor-based active measurements and passive data related to disease symptoms and severity in patients with MS [23,31-35]. In contrast to previous studies, elevateMS collected data from a large, geographically diverse, remote, and unsupervised population, independent of scheduled clinic visits. Although a significant number of individuals did not participate beyond enrollment on the first day of the elevateMS study, our user engagement data are consistent with previous digital health studies that recruited broadly from the general population, with no scheduled in-clinic touchpoints or incentives associated with the app usage [62]. As with previous digital health studies [26,28,34], elevateMS relied on arbitrary measures of retention and compliance. In order to fully assess participant engagement and enable comparisons between different studies, these parameters need to be defined in more specific terms. For example, the BEST (Biomarkers, EndpointS, and other Tools) Resource, created by the US Food and Drug Administration and the National Institutes of Health [80], could be expanded to include clear and unambiguous definitions of retention and compliance in digital health studies, creating standardized measures that could be utilized across the field.

Clinical validation of elevateMS data was beyond the scope of this study; however, it is reassuring that the symptoms and triggers most commonly reported in the app, such as fatigue, weakness, temperature and stress, are already well-documented in traditional studies of MS [7-10,81]. This is further reinforced by our results showing worse performance in active functional tests, such as finger-tapping and DSST responses, in increased ambient temperatures, reflecting the well-known heat sensitivity experienced by patients with MS [7-9]. Furthermore, by comparing self-reported PDDS and Neuro-QoL results, we have shown that it is possible to use smartphone-based motor measurements to assess both disease severity and QoL, providing internal validation of elevateMS results.

### Conclusions

In contrast to current, episodic disease monitoring practices, this study demonstrates the value and utility of frequently assessing the real-world, live patient experience of MS using a digital health app. By providing a more comprehensive and representative assessment of patients outside of the clinic, elevateMS and other disease-tracking apps have the potential to enhance the understanding of MS, facilitate patient–clinician communication, and support personalization of disease management plans.

## Appendix

Multimedia Appendix 1 Overview of user-centered design process used to create the elevateMS study app.

Multimedia Appendix 2 Example elevateMS survey questions.

Multimedia Appendix 3 Example features from active functional performance tests.

Multimedia Appendix 4 Missing responses in baseline sociodemographic characteristics of study participants.

Multimedia Appendix 5 Summary of activity-specific compliance across the 12-week study duration.

Multimedia Appendix 6 Distribution of overall activity type per user for participants with multiple sclerosis.

Multimedia Appendix 7 Participant-reported symptoms and triggers.

Multimedia Appendix 8 Example assessment of participant performance in the walk and balance sensor-based active functional test.

Multimedia Appendix 9 Association between baseline characteristics and active functional test performance in participants with MS (top features for each test).

Multimedia Appendix 10 Heatmap showing association between all recorded baseline characteristics in participants with multiple sclerosis.

Multimedia Appendix 11 Association between Neuro-QoL™ domains and functional test performance in participants with MS (top features for each test).

Multimedia Appendix 12 Association between local weather conditions and functional test performance in participants with MS.

Multimedia Appendix 13 Association between local weather conditions and PROs in participants with MS.

Multimedia Appendix 14 Association between local weather conditions and PROs in participants with MS (sensitivity analysis).

## Abbreviations

DMT: disease-modifying therapy
DSST: Digit Symbol Substitution Test
LME: linear mixed effects
MS: multiple sclerosis
Neuro-QoL: Quality of Life in Neurological Disorders
PDDS: Patient-Determined Disease Steps
PRO: patient-reported outcome
QoL: quality of life
