# Supplementary material for: Evaluating the Utility of Smartphone-Based Sensor Assessments in Persons With Multiple Sclerosis in the Real-World Using an App (elevateMS): Observational, Prospective Pilot Digital Health Study
Source: JMIR Mhealth Uhealth. 2020 Oct 27;8(10):e22108. doi: 10.2196/22108 (PMC7655470; doi:10.2196/22108)
Supplement: Multimedia Appendix 1 [file mhealth_v8i10e22108_app1.docx]

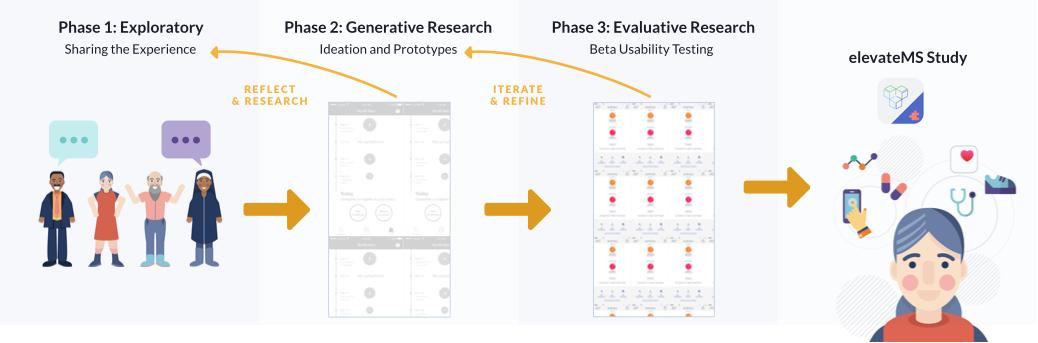


**Multimedia Appendix 1.** Overview of user-centered design process used to create the elevateMS study app. The patient-centered design process for the elevateMS study app consisted of three phases. **Phase 1** was exploratory and involved semi-structured interviews with 10 patient advisors with MS, who were recruited through a Health Advocacy Group (<http://hastrategies.com/>) and compensated with $25 for their time. The interviews were used understand the advisors’ perspective of living with MS by investigating: 1) how individuals manage their unique daily encounters with the disease and what quality of life with MS means to them; 2) their expectations of using a mobile application (app) designed to quantify their symptoms via research assessments and 3) any particular app features they observe that might fit naturally into their daily routine with MS. **Phase 2** was generative and involved translating the key needs identified from Phase 1 into low-fidelity app prototypes. Advisors downloaded the prototype onto their smartphone and participated in Think-Aloud and Task Analysis exercises to evaluate the user interface. This phase included multiple iterations of the app to test and refine the design and address concerns related to unclear instructions and/or confusing illustrations. **Phase 3** was evaluative and used the results from Phase 2 to create a beta app for testing. Advisors were observed while registering and using the elevateMS app for the first time during a 1-hour Think Aloud exercise. Advisors continued to use the app for a week during the course of daily life and provided feedback during a 1-hour follow-up session regarding participant burden, instructions, and overall usability in order to refine elevateMS before the broader launch.
