# Supplementary material for: Evaluating the Utility of Smartphone-Based Sensor Assessments in Persons With Multiple Sclerosis in the Real-World Using an App (elevateMS): Observational, Prospective Pilot Digital Health Study
Source: JMIR Mhealth Uhealth. 2020 Oct 27;8(10):e22108. doi: 10.2196/22108 (PMC7655470; doi:10.2196/22108)
Supplement: Multimedia Appendix 2 [file mhealth_v8i10e22108_app2.docx]

**Multimedia Appendix 2.** Example elevateMS survey questions.

| **Question** | **Possible responses** |
| --- | --- |
| **Overall physical ability** | |
| Patient-Determined Disease Steps^a^ | *Select one option:*   - - Normal (*I have some mild symptoms, mostly sensory due to MS, but they do not limit my activity. If I do have an attack, I return to normal when the attack has passed)*   - Mild disability (*I have some noticeable symptoms from my MS, but they are minor and have only a small effect on my lifestyle)*   - Moderate disability (*I don’t have any limitations in my walking ability. However, I do have significant problems due to MS that limit daily activities in other ways)*   - Gait disability (*MS does interfere with my activities, especially my walking. I can work a full day, but athletic or physically demanding activities are more difficult than they used to be. I usually don’t need a cane or other assistance to walk, but I might need some assistance during an attack)* |
| **Neuro-QoL™ short-form: Cognitive Function domain** | |
| - In the past 7 days:   - I had to read something several times to understand it   - My thinking was slow   - I had to work really hard to pay attention or I would make a mistake   - I had trouble concentrating - How much difficulty do you currently have:   - Reading and following complex instructions (eg, directions for a new medication)?   - Planning for and keeping appointments that are not part of your weekly routine (eg, a therapy or doctor appointment, or a social gathering with friends and family)?   - Managing your time to do most of your daily activities?   - Learning new tasks or instructions? | *For each question, select from 1–5 scale:*  *1 – Never*  *2 – Rarely (once)*  *3 – Sometimes (2–3 times)*  *4 – Often (once a day)*  *5 – Very often (several times a day)*  *For each question, select from 1–5 scale:*  *1 – None*  *2 – A little*  *3 – Somewhat*  *4 – A lot*  *5 – Cannot do* |
| **Neuro-QoL™ short-form: Upper Extremity Function domain** | |
| - Are you able to turn a key in a lock? - Are you able to brush your teeth? - Are you able to make a phone call using a touch tone keypad? - Are you able to pick up coins from a table top? - Are you able to write with a pen or pencil? - Are you able to open and close a zipper? - Are you able to wash and dry your body? - Are you able to shampoo your hair? | *For each question, select from 1–5 scale:*  *1 – Without any difficulty*  *2 – With a little difficulty*  *3 – With some difficulty*  *4 – With much difficulty*  *5 – Unable to do* |
| **Neuro-QoL™ short-form: Lower Extremity Function domain** | |
| - Are you able to get on and off the toilet? - Are you able to step up and down curbs? - Are you able to get in and out of a car? - Are you able to get out of bed into a chair? - Are you able to push open a heavy door? - Are you able to run errands and shop? - Are you able to get up off the floor from lying on your back without help? - Are you able to go for a walk of at least 10 minutes? | *For each question, select from 1–5 scale:*  *1 – Without any difficulty*  *2 – With a little difficulty*  *3 – With some difficulty*  *4 – With much difficulty*  *5 – Unable to do* |

^a^Truncated 4-point scale. MS, multiple sclerosis; Neuro-QoL™, Quality of Life in Neurological Disorders.
