# Supplementary material for: Evaluating the Utility of Smartphone-Based Sensor Assessments in Persons With Multiple Sclerosis in the Real-World Using an App (elevateMS): Observational, Prospective Pilot Digital Health Study
Source: JMIR Mhealth Uhealth. 2020 Oct 27;8(10):e22108. doi: 10.2196/22108 (PMC7655470; doi:10.2196/22108)
Supplement: Multimedia Appendix 3 [file mhealth_v8i10e22108_app3.docx]

**Multimedia Appendix 3.** Example features from active functional performance tests.

| **Active functional performance test** | **Data captured** | **Number of default features** | **Key features** |
| --- | --- | --- | --- |
| Finger-tapping | Positions;  timestamps of taps | 42 | Number of taps in 20 seconds;  variation in tapping frequency;  tapping location drift |
| Walk | Accelerometer;  gyroscope;  magnetometer;  number of steps | 114 | Heal strike frequency;  variation in heal strike frequency |
| Balance | Accelerometer;  gyroscope;  magnetometer | 19 | Sway;  fundamental frequency |
| Finger-to-nose | Accelerometer;  gyroscope;  magnetometer | 100 | Fundamental frequency;  displacement around path |
