# Supplementary material for: Evaluating the Utility of Smartphone-Based Sensor Assessments in Persons With Multiple Sclerosis in the Real-World Using an App (elevateMS): Observational, Prospective Pilot Digital Health Study
Source: JMIR Mhealth Uhealth. 2020 Oct 27;8(10):e22108. doi: 10.2196/22108 (PMC7655470; doi:10.2196/22108)
Supplement: Multimedia Appendix 4 [file mhealth_v8i10e22108_app4.docx]

**Multimedia Appendix 4.** Missing responses in baseline sociodemographic characteristics of study participants.

| **Characteristic, n (%)** | **Controls**  (n=134) | **Participants with MS**  (self-referred;  n=359) | **Participants with MS**  (clinic-referred;  n=136) |
| --- | --- | --- | --- |
| Gender | 92 (68.7) | 149 (41.5) | 44 (32.4) |
| Race | 90 (67.2) | 146 (40.7) | 44 (32.4) |
| Education | 91 (67.9) | 145 (40.4) | 45 (33.1) |
| Health insurance | 91 (67.9) | 146 (40.7) | 44 (32.4) |
| Employment status | 91 (67.9) | 145 (40.4) | 44 (32.4) |

Missing indicates where responses were not provided by participants. MS, multiple sclerosis.
