# Supplementary material for: Evaluating the Utility of Smartphone-Based Sensor Assessments in Persons With Multiple Sclerosis in the Real-World Using an App (elevateMS): Observational, Prospective Pilot Digital Health Study
Source: JMIR Mhealth Uhealth. 2020 Oct 27;8(10):e22108. doi: 10.2196/22108 (PMC7655470; doi:10.2196/22108)
Supplement: Multimedia Appendix 5 [file mhealth_v8i10e22108_app5.docx]

**Multimedia Appendix 5.** Summary of activity-specific compliance across the 12-week study duration.

|  | **PRO** | | | | **Active functional test** | | | |
| --- | --- | --- | --- | --- | --- | --- | --- | --- |
| **Number of unique participants (total number of times activity was completed by MS participants)^a^** | **Daily check-in^b^** | **Symptoms** | **Triggers** | **Neuro-QoL™** | **Finger-tapping** | **Walk and balance** | **DSST** | **Finger-to-nose** |
| Week 1 | 372 (1149) | 381 (851) | 353 (765) | 44 (99) | 398 (586) | 225 (301) | 298 (415) | 347 (490) |
| Week 2 | 179 (653) | 125 (361) | 107 (306) | 54 (111) | 177 (237) | 111 (146) | 133 (175) | 163 (221) |
| Week 3 | 150 (584) | 96 (305) | 85 (258) | 75 (157) | 149 (186) | 86 (106) | 108 (132) | 134 (162) |
| Week 4 | 136 (520) | 80 (241) | 69 (197) | 47 (96) | 121 (164) | 79 (106) | 89 (113) | 107 (138) |
| Week 5 | 114 (469) | 68 (207) | 59 (173) | 49 (107) | 106 (140) | 71 (109) | 76 (98) | 98 (132) |
| Week 6 | 105 (413) | 50 (177) | 45 (138) | 42 (90) | 92 (126) | 67 (95) | 72 (91) | 85 (117) |
| Week 7 | 99 (364) | 52 (155) | 43 (115) | 25 (56) | 82 (104) | 56 (77) | 59 (74) | 77 (96) |
| Week 8 | 94 (383) | 51 (151) | 41 (124) | 42 (86) | 88 (116) | 51 (74) | 67 (84) | 81 (102) |
| Week 9 | 92 (357) | 51 (170) | 43 (129) | 41 (77) | 76 (101) | 49 (70) | 60 (74) | 73 (94) |
| Week 10 | 84 (304) | 39 (116) | 33 (95) | 32 (63) | 67 (86) | 45 (61) | 50 (63) | 65 (82) |
| Week 11 | 86 (322) | 40 (120) | 32 (104) | 31 (56) | 65 (91) | 39 (49) | 46 (59) | 62 (80) |
| Week 12 | 72 (288) | 31 (119) | 30 (94) | 32 (59) | 60 (85) | 37 (52) | 47 (58) | 57 (73) |

^a^For each activity type, the total number of unique participants who completed the activity each week is reported, with the total number of times the activity was completed by MS participants listed in brackets; ^b^Daily health, mobility and pain survey. As the frequency of data collection for each of these activities differed significantly based on the underlying study protocol (see **Table 1** in main text), comparisons should not be made across different activities, but instead for each individual activity over time. DSST, Digit Symbol Substitution Test; Neuro-QoL™; Quality of Life in Neurological Disorders; PROs, patient-reported outcomes.
