# Supplementary figures and images for: Evaluating the Utility of Smartphone-Based Sensor Assessments in Persons With Multiple Sclerosis in the Real-World Using an App (elevateMS): Observational, Prospective Pilot Digital Health Study
Source: JMIR Mhealth Uhealth. 2020 Oct 27;8(10):e22108. doi: 10.2196/22108 (PMC7655470; doi:10.2196/22108)

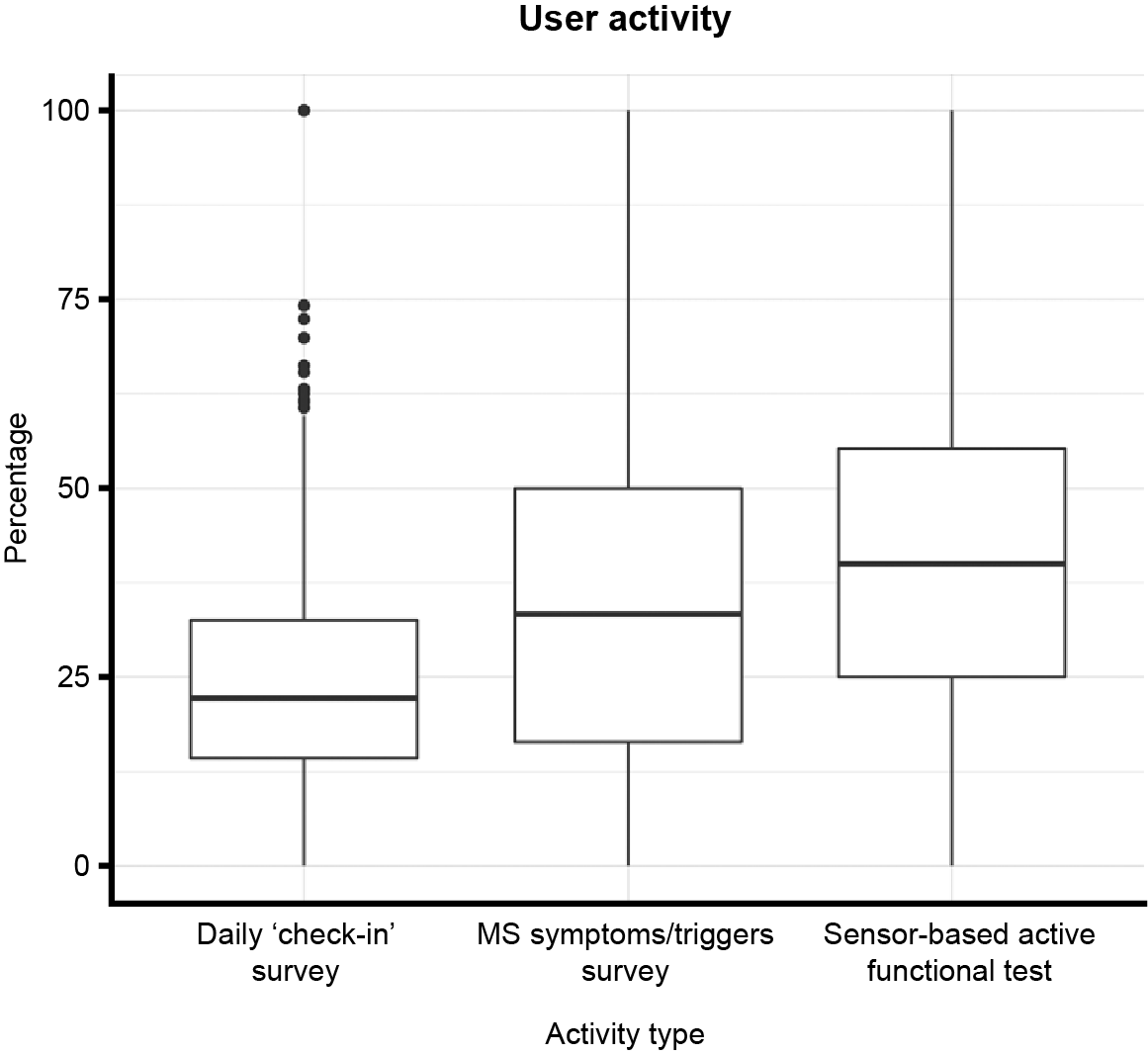

Supplement: Multimedia Appendix 6 [file mhealth_v8i10e22108_app6.png]
