# Supplementary material for: Evaluating the Utility of Smartphone-Based Sensor Assessments in Persons With Multiple Sclerosis in the Real-World Using an App (elevateMS): Observational, Prospective Pilot Digital Health Study
Source: JMIR Mhealth Uhealth. 2020 Oct 27;8(10):e22108. doi: 10.2196/22108 (PMC7655470; doi:10.2196/22108)
Supplement: Multimedia Appendix 7 [file mhealth_v8i10e22108_app7.docx]

**Multimedia Appendix 7.** Participant-reported symptoms and triggers.

|  | **Proportion of participants with MS** |
| --- | --- |
| **Symptom, n (%)** | |
| Fatigue | 310 (76.9) |
| Weakness | 222 (55.1) |
| Memory and attention issues | 209 (51.9) |
| Walking difficulties | 205 (50.9) |
| Brain fog | 200 (49.6) |
| Challenges with balance | 199 (49.4) |
| Stiff muscles | 195 (48.4) |
| Bladder problems | 190 (47.1) |
| Pins and needles sensation | 190 (47.1) |
| Clumsiness | 185 (45.9) |
| Problems with coordination | 158 (39.2) |
| Arm and or leg | 153 (38.0) |
| Anxiety | 149 (37.0) |
| Depression | 149 (37.0) |
| Back pain | 145 (36.0) |
| Dizziness and vertigo | 141 (35.0) |
| Cramping | 139 (34.5) |
| Headache | 138 (34.2) |
| Vision problems | 136 (33.7) |
| Reduced sensation of touch | 135 (33.5) |
| Overall muscle and nerve pain | 132 (32.8) |
| Uncomfortable tingling | 115 (28.5) |
| Mood swings | 109 (27.0) |
| Itching | 106 (26.3) |
| Bowel problems | 105 (26.1) |
| Burning sensation | 94 (23.3) |
| Tremor | 94 (23.3) |
| Speech problems | 93 (23.1) |
| Sexual problems | 83 (20.6) |
| Swallowing problems | 63 (15.6) |
| Eye pain | 54 (13.4) |
| Hearing loss | 49 (12.2) |
| Abnormality of taste | 39 (9.7) |
| Breathing problems | 28 (6.9) |
| Tongue numbness | 26 (6.5) |
| Seizures | 11 (2.7) |
| **Trigger, n (%)** | |
| Hot temperature | 259 (69.6) |
| Stress | 250 (67.2) |
| Late bedtime | 221 (59.4) |
| Humid | 171 (46.0) |
| Exercise | 168 (45.2) |
| Illness | 145 (39.0) |
| Cold temperature | 127 (34.1) |
| Diet | 125 (33.6) |
| Early bedtime | 97 (26.1) |
| Rain | 97 (26.1) |
| Lifestyle | 93 (25.0) |
| Travelling | 91 (24.5) |

Percentages were calculated based on the total number of participants with MS who provided a response (symptoms, n=403; triggers, n=372). MS, multiple sclerosis.
