# Supplementary material for: Evaluating the Utility of Smartphone-Based Sensor Assessments in Persons With Multiple Sclerosis in the Real-World Using an App (elevateMS): Observational, Prospective Pilot Digital Health Study
Source: JMIR Mhealth Uhealth. 2020 Oct 27;8(10):e22108. doi: 10.2196/22108 (PMC7655470; doi:10.2196/22108)
Supplement: Multimedia Appendix 8 [file mhealth_v8i10e22108_app8.docx]

**Multimedia Appendix 8.** Example assessment of participant performance in the walk and balance sensor-based active functional test.


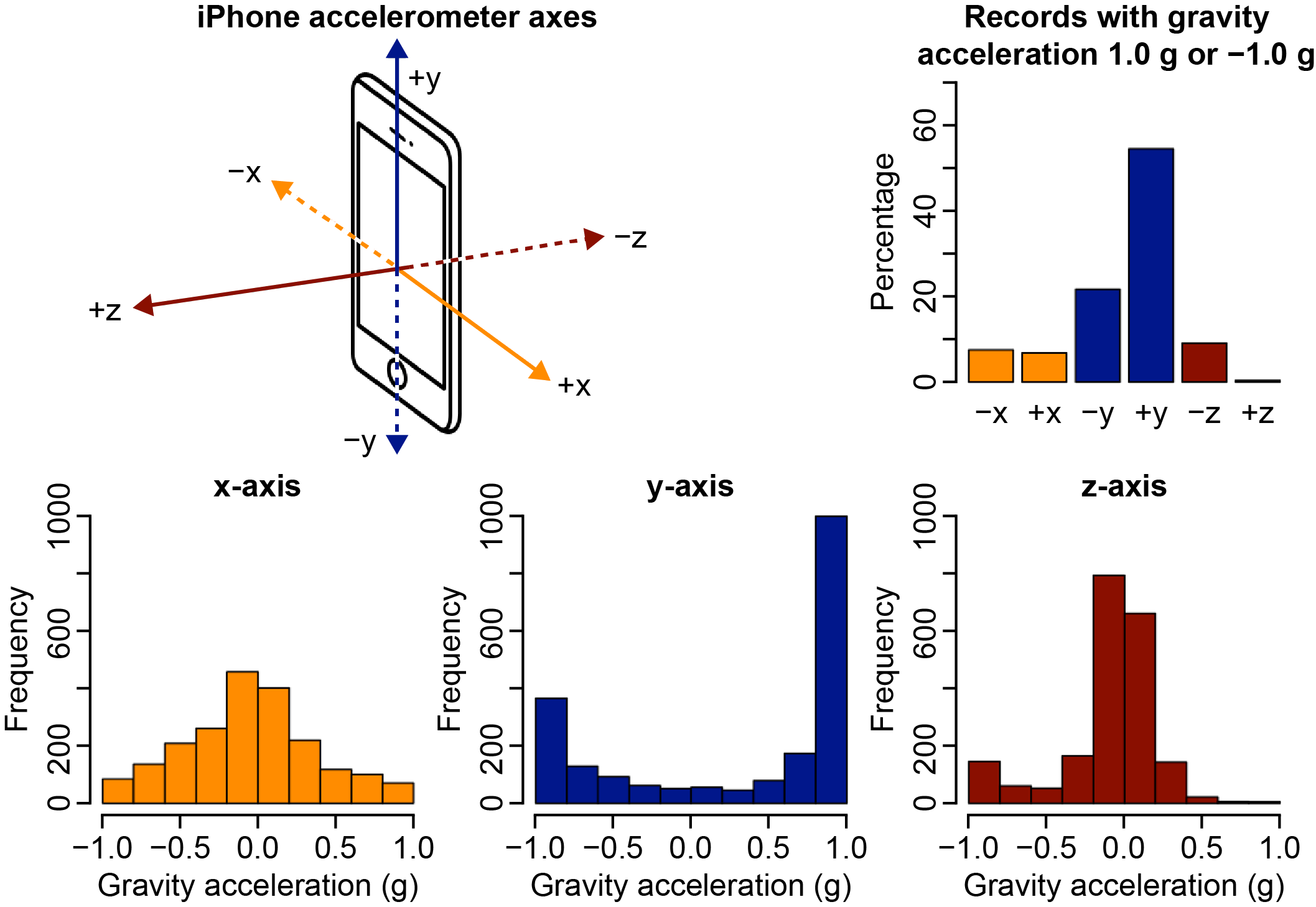


The iPhone accelerometer measures device acceleration in each of the three axes shown (x, y and z). The values reported by the accelerometer are measured in increments of the gravitational acceleration, with the value 1.0 g representing an acceleration of 9.8 m/s^−2^in the given direction. The direction of the acceleration can be used to indicate if a participant carried out an active test correctly. The top right graph shows the proportion of records for the walk and balance test with gravity acceleration values of either 1.0 g or −1.0 g in each axis. In this test, participants were required to walk for 20 seconds then stand still for 10 seconds with their iPhone in their pocket in order to assess gait, posture, stability, and balance. Overall, 76.2% of records show the strongest gravity accelerations along the y-axis (21.7% for −y and 54.5% for +y), which suggests that the participants followed the instructions and kept the phone in their pocket. However, 9.49% of the recordings were closer to the −z-axis, which suggests that participants were holding the phone in their hand with the screen pointing towards the ﬂoor. In addition, 14.26% of the recordings were associated with the x-axis, which either suggests they had large pockets where the phone sat horizontally or that participants were holding the phone in their hand with the screen either facing their leg (−x) or facing outwards (+x). The three bottom histograms show the frequency of gravitational acceleration values along the x-, y- and z-axes.
