# Supplementary material for: Evaluating the Utility of Smartphone-Based Sensor Assessments in Persons With Multiple Sclerosis in the Real-World Using an App (elevateMS): Observational, Prospective Pilot Digital Health Study
Source: JMIR Mhealth Uhealth. 2020 Oct 27;8(10):e22108. doi: 10.2196/22108 (PMC7655470; doi:10.2196/22108)
Supplement: Multimedia Appendix 9 [file mhealth_v8i10e22108_app9.docx]

**Multimedia Appendix 9.** Association between baseline characteristics and active functional test performance in participants with MS (top features for each test).

| **Baseline characteristic** | **Category** | **Feature** | **Effect size** | ***P* value_mixed model_** | ***P* value_ANOVA (mixed model)_** |
| --- | --- | --- | --- | --- | --- |
| **Finger-tapping** |  |  |  |  |  |
| Overall physical ability^a^ | Mild | numberTaps.median | –13.871 | .001 | <.001 |
|  | Moderate | numberTaps.median | –40.699 | <.001 | <.001 |
|  | Gait | numberTaps.median | –43.635 | <.001 | <.001 |
| Age group, years | 30–39 | numberTaps.median | –5.433 | .472 | <.001 |
|  | 40–49 | numberTaps.median | –8.194 | .271 | <.001 |
|  | 50–59 | numberTaps.median | –28.613 | <.001 | <.001 |
|  | 60+ | numberTaps.median | –40.642 | <.001 | <.001 |
| Education | High school diploma/GED | numberTaps.median | –12.801 | .088 | .001 |
|  | Postgraduate degree | numberTaps.median | 18.608 | <.001 | .001 |
|  | Other | numberTaps.median | –13.581 | .376 | .001 |
| Race | Non-Caucasian | numberTaps.median | –3.101 | .568 | .694 |
| Years since first DMT | 4–10 | numberTaps.median | –3.249 | .487 | .004 |
|  | 10–18 | numberTaps.median | –12.277 | .009 | .004 |
|  | >18 | numberTaps.median | –22.160 | <.001 | .004 |
| Years since diagnosis | 4–10 | numberTaps.median | –2.873 | .552 | .009 |
|  | 10–18 | numberTaps.median | –14.406 | .003 | .009 |
|  | >18 | numberTaps.median | –19.055 | <.001 | .009 |
| MS family history | Yes | numberTaps.median | 5.342 | .222 | .464 |
|  | Not sure | numberTaps.median | –15.259 | .022 | .464 |
| **Walk and balance** |  |  |  |  |  |
| Overall physical ability^a^ | Mild | medianF0FAJ.median^b^ | –0.082 | .242 | .001 |
|  | Moderate | medianF0FAJ.median^b^ | –0.169 | .030 | .001 |
|  | Gait | medianF0FAJ.median^b^ | –0.387 | <.001 | .001 |
| Age group, years | 30–39 | medianF0FAJ.median^b^ | –0.117 | .412 | .921 |
|  | 40–49 | medianF0FAJ.median^b^ | –0.035 | .801 | .921 |
|  | 50–59 | medianF0FAJ.median^b^ | –0.110 | .429 | .921 |
|  | 60+ | medianF0FAJ.median^b^ | –0.072 | .626 | .921 |
| Education | High school diploma/GED | medianF0FAJ.median^b^ | 0.049 | .656 | .921 |
|  | Postgraduate degree | medianF0FAJ.median^b^ | 0.060 | .372 | .921 |
|  | Other | medianF0FAJ.median^b^ | –0.038 | .859 | .921 |
| Race | Non-Caucasian | medianF0FAJ.median^b^ | –0.066 | .453 | .976 |
| Years since first DMT | 4–10 | medianF0FAJ.median^b^ | 0.022 | .778 | .284 |
|  | 10–18 | medianF0FAJ.median^b^ | 0.129 | .087 | .284 |
|  | >18 | medianF0FAJ.median^b^ | –0.067 | .424 | .284 |
| Years since diagnosis | 4–10 | medianF0FAJ.median^b^ | 0.025 | .748 | .541 |
|  | 10–18 | medianF0FAJ.median^b^ | 0.144 | .068 | .541 |
|  | >18 | medianF0FAJ.median^b^ | –0.029 | .717 | .541 |
| MS family history | Yes | medianF0FAJ.median^b^ | 0.112 | .101  0.436 | .664 |
|  | Not Sure | medianF0FAJ.median^b^ | –0.087 | .436 | .664 |
| **DSST** |  |  |  |  |  |
| Overall physical ability^a^ | Mild | numCorrect.median | –1.216 | .392 | .005 |
|  | Moderate | numCorrect.median | –4.773 | .002 | .005 |
|  | Gait | numCorrect.median | –5.468 | <.001 | .005 |
| Age group, years | 30–39 | numCorrect.median | –4.269 | .090 | .224 |
|  | 40–49 | numCorrect.median  kkkkknumCorrect.median | –4.526 | .067 | .224 |
|  | 50–59 | numCorrect.median | –6.480 | .009 | .224 |
|  | 60+ | numCorrect.median | –7.260 | .006 | .224 |
| Education | High school diploma/GED | numCorrect.median | –3.952 | .077 | .330 |
|  | Postgraduate degree | numCorrect.median | 0.873 | .520 | .330 |
|  | Other | numCorrect.median | –4.752 | .472 | .330 |
| Race | Non-Caucasian | numCorrect.median | –4.888 | .006 | .012 |
| Years since first DMT | 4–10 | numCorrect.median | 0.184 | .903 | .793 |
|  | 10–18 | numCorrect.median | –0.942 | .519 | .793 |
|  | >18 | numCorrect.median | –1.146 | .497 | .793 |
| Years since diagnosis | 4–10 | numCorrect.median | 0.025 | .987 | .891 |
|  | 10–18 | numCorrect.median | –0.966 | .527 | .891 |
|  | >18 | numCorrect.median | –0.685 | .664 | .891 |
| MS family history | Yes | numCorrect.median | –0.119 | .931 | .956 |
|  | Not sure | numCorrect.median | –1.511 | .500 | .956 |
| **Finger-to-nose** |  |  |  |  |  |
| Overall physical ability^a^ | Mild | skewness.tm.IMF1.md_uav_gyroscope.IQR^c^ | –0.003 | .434 | .010 |
|  | Moderate | skewness.tm.IMF1.md_uav_gyroscope.IQR^c^ | 0.007 | .096 | .010 |
|  | Gait | skewness.tm.IMF1.md_uav_gyroscope.IQR^c^ | 0.013 | <.001 | .010 |
| Age group, years | 30–39 | skewness.tm.IMF1.md_uav_gyroscope.IQR^c^ | 0.003 | .695 | .125 |
|  | 40–49 | skewness.tm.IMF1.md_uav_gyroscope.IQR^c^ | 0.009 | .174 | .125 |
|  | 50–59 | skewness.tm.IMF1.md_uav_gyroscope.IQR^c^ | 0.008 | .205 | .125 |
|  | 60+ | skewness.tm.IMF1.md_uav_gyroscope.IQR^c^ | 0.016 | .018 | .125 |
| Education | High school diploma/GED | skewness.tm.IMF1.md_uav_gyroscope.IQR^c^ | 0.009 | .078 | .059 |
|  | Postgraduate degree | skewness.tm.IMF1.md_uav_gyroscope.IQR^c^ | 0.006 | .044 | .059 |
|  | Other | skewness.tm.IMF1.md_uav_gyroscope.IQR^c^ | –0.021 | .032 | .059 |
| Race | Non-Caucasian | skewness.tm.IMF1.md_uav_gyroscope.IQR^c^ | –0.002 | .584 | .876 |
| Years since first DMT | 4–10 | skewness.tm.IMF1.md_uav_gyroscope.IQR^c^ | 0.003 | .413 | .500 |
|  | 10–18 | skewness.tm.IMF1.md_uav_gyroscope.IQR^c^ | 0.001 | .708 | .500 |
|  | >18 | skewness.tm.IMF1.md_uav_gyroscope.IQR^c^ | 0.007 | .064 | .500 |
| Years since diagnosis | 4–10 | skewness.tm.IMF1.md_uav_gyroscope.IQR^c^ | 0.003 | .427 | .658 |
|  | 10–18 | skewness.tm.IMF1.md_uav_gyroscope.IQR^c^ | 0.003 | .418 | .658 |
|  | >18 | skewness.tm.IMF1.md_uav_gyroscope.IQR^c^ | 0.006 | .104 | .658 |
| MS family history | Yes | skewness.tm.IMF1.md_uav_gyroscope.IQR^c^ | –0.002 | .493 | .771 |
|  | Not sure | skewness.tm.IMF1.md_uav_gyroscope.IQR^c^ | 0.004 | .456 | .771 |

^a^Based on truncated 4-point Patient-Determined Disease Steps scale; ^b^F0FAJ represents frequency at which the maximum peak of the Lomb-Scargle periodogram occurred for the average acceleration series, with frequencies limited to 0.2–5 Hz. ^c^skewness.tm.IMF1.md_uav_gyroscope.IQR represents hand rotation velocity feature derived from device gyroscope. All results were analyzed using a linear mixed-effects model (*P* value_mixed model_) followed by ANOVA (*P* value_ANOVA (mixed model)_). ANOVA, analysis of variance; DMT, disease-modifying therapy; GED, General Educational Development; MS, multiple sclerosis.
