# Supplementary material for: Evaluating the Utility of Smartphone-Based Sensor Assessments in Persons With Multiple Sclerosis in the Real-World Using an App (elevateMS): Observational, Prospective Pilot Digital Health Study
Source: JMIR Mhealth Uhealth. 2020 Oct 27;8(10):e22108. doi: 10.2196/22108 (PMC7655470; doi:10.2196/22108)
Supplement: Multimedia Appendix 10 [file mhealth_v8i10e22108_app10.docx]

**Multimedia Appendix 10.** Heatmap showing association between all recorded baseline characteristics in participants with MS.


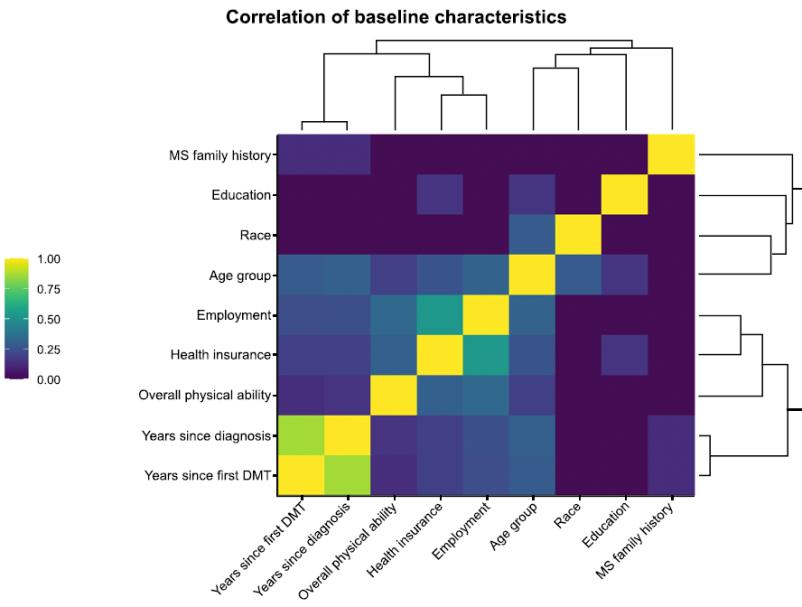


| **Baseline characteristic** | ***P* value** | **Correlation coefficient  (versus overall physical ability)** |
| --- | --- | --- |
| Overall physical ability^a^ | <.001 | 1.0000 |
| Years since first DMT | .003 | 0.1299 |
| Years since diagnosis | <.001 | 0.1473 |
| Health insurance | <.001 | 0.3005 |
| Employment status | <.001 | 0.3374 |
| Age group | <.001 | 0.1851 |
| Race | .434 | 0.0947 |
| Education | .591 | 0.0902 |
| MS family history | .205 | 0.0927 |

^a^Based on 4-point Patient-Determined Disease Steps scale. Spearman correlation was used for assessing association between continuous variables, while Cramer’s V determined from Pearson chi-squared test was used for testing association between factor variables. DMT, disease modifying therapy; MS, multiple sclerosis.
