# Supplementary material for: Evaluating the Utility of Smartphone-Based Sensor Assessments in Persons With Multiple Sclerosis in the Real-World Using an App (elevateMS): Observational, Prospective Pilot Digital Health Study
Source: JMIR Mhealth Uhealth. 2020 Oct 27;8(10):e22108. doi: 10.2196/22108 (PMC7655470; doi:10.2196/22108)
Supplement: Multimedia Appendix 11 [file mhealth_v8i10e22108_app11.docx]

**Multimedia Appendix 11.** Association between Neuro-QoL™ domains and functional test performance in participants with MS (top features for each test).

|  |  |  |  |  |  |  | **Sensitivity analysis** | | | |
| --- | --- | --- | --- | --- | --- | --- | --- | --- | --- | --- |
| **Neuro-QoL™ domain** | **Neuro-QoL™ category** | **Feature** | **Effect size** | **Standard error** | ***P* value_mixed model_** | ***P* value_ANOVA (mixed model)_** | **Effect size** | **Standard error** | ***P* value_mixed model_** | ***P* value_ANOVA (mixed model)_** |
| **Finger-tapping** |  |  |  |  |  |  |  |  |  |  |
| Upper Extremity Function | Mild | maxTapInter | 0.152 | 0.038 | <.001 | <.001 | 0.152 | 0.038 | <.001 | <.001 |
|  | Moderate | maxTapInter | 0.404 | 0.071 | <.001 | <.001 | 0.404 | 0.071 | <.001 | <.001 |
| **Walk and balance** |  |  |  |  |  |  |  |  |  |  |
| Lower Extremity Function | Mild | P0Y^a^ | ‒67.262 | 20.581 | .001 | .015 | ‒67.261 | 20.581 | .001 | .014 |
|  | Moderate | P0Y^a^ | ‒99.181 | 25.142 | <.001 | .015 | ‒99.180 | 25.141 | <.001 | .014 |
| **DSST** |  |  |  |  |  |  |  |  |  |  |
| Cognitive Function | Mild | sdTime | 0.143 | 0.379 | .706 | .027 | .203 | .294 | .490 | .886 |
|  | Moderate | sdTime | 0.302 | 0.414 | .466 | .027 | .355 | .315 | .261 | .886 |
|  | Severe | sdTime | 1.600 | 0.530 | .003 | .027 | - | - | - | - |
| Lower Extremity Function | Mild | sdTime | 0.310 | 0.241 | .201 | <.001 | 0.318 | 0.232 | .174 | .440 |
|  | Moderate | sdTime | 0.104 | 0.275 | .706 | <.001 | 0.083 | 0.266 | .755 | .440 |
|  | Severe | sdTime | 10.309 | 1.084 | <.001 | <.001 | - | - | - | - |
| **Finger-to-nose** |  |  |  |  |  |  |  |  |  |  |
| Upper Extremity Function | Mild | skewness.tm.IMF1.iqr_uaa_gyroscope^b^ | 0.037 | 0.013 | .004 | .003 | 0.037 | 0.013 | .004 | .003 |
|  | Moderate | skewness.tm.IMF1.iqr_uaa_gyroscope^b^ | 0.105 | 0.024 | <.001 | .003 | 0.105 | 0.024 | <.001 | .003 |

^a^P0Y represents maximum power in the inspected frequency interval of the Lomb-Scargle periodogram for the Y acceleration series; ^b^skewness.tm.IMF1.iqr_uaa_gyroscope represents hand rotation acceleration feature derived from device gyroscope. Effect size shows the correlation between each active functional test feature and Neuro-QoL™ category and was calculated using the Normal Neuro-QoL™ category as baseline. All results were analyzed using a linear mixed-effects model (*P* value_mixed model_) followed by ANOVA (*P* value_ANOVA (mixed model)_). Additional sensitivity analyses were conducted to evaluate the impact of the extreme Neuro-QoL™ category (Severe) on the association between Neuro-QoL™ and functional tests. ANOVA, analysis of variance; DSST, Digit Symbol Substitution Test; MS, multiple sclerosis; Neuro-QoL™; Quality of Life in Neurological Disorders; sd, standard deviation.
