# Supplementary material for: Evaluating the Utility of Smartphone-Based Sensor Assessments in Persons With Multiple Sclerosis in the Real-World Using an App (elevateMS): Observational, Prospective Pilot Digital Health Study
Source: JMIR Mhealth Uhealth. 2020 Oct 27;8(10):e22108. doi: 10.2196/22108 (PMC7655470; doi:10.2196/22108)
Supplement: Multimedia Appendix 12 [file mhealth_v8i10e22108_app12.docx]

**Multimedia Appendix 12.** Association between local weather conditions and functional test performance in participants with MS.

| **Weather parameter** | **Feature** | **Effect size** | ***P* value** |
| --- | --- | --- | --- |
| **Finger-tapping** |  | | |
| Current temperature (°F) | numberTaps | ‒0.142 | <.001 |
| Minimum temperature (°F) | numberTaps | ‒0.155 | <.001 |
| Maximum temperature (°F) | numberTaps | ‒0.129 | <.001 |
| Relative humidity (%) | numberTaps | ‒0.001 | .972 |
| Cloud coverage (%) | numberTaps | 0.002 | .925 |
| Atmospheric pressure (hPa) | numberTaps | ‒0.019 | .859 |
| **DSST** |  | | |
| Current temperature (°F) | numCorrect | ‒0.056 | .009 |
| Minimum temperature (°F) | numCorrect | ‒0.053 | .011 |
| Maximum temperature (°F) | numCorrect | ‒0.055 | .009 |
| Relative humidity (%) | numCorrect | ‒0.001 | .981 |
| Cloud coverage (%) | numCorrect | 0.005 | .544 |
| Atmospheric pressure (hPa) | numCorrect | ‒0.032 | .544 |
| **Finger-to-nose** |  | | |
| Current temperature (°F) | energy.tm.IMF1.md_uj_accelerometer^a^ | ‒53.883 | <.001 |
| Minimum temperature (°F) | energy.tm.IMF1.md_uj_accelerometer^a^ | ‒55.988 | <.001 |
| Maximum temperature (°F) | energy.tm.IMF1.md_uj_accelerometer^a^ | ‒50.765 | <.001 |
| Relative humidity (%) | energy.tm.IMF1.md_uj_accelerometer^a^ | ‒12.154 | .095 |
| Cloud coverage (%) | energy.tm.IMF1.md_uj_accelerometer^a^ | ‒5.469 | .173 |
| Atmospheric pressure (hPa) | energy.tm.IMF1.md_uj_accelerometer^a^ | 30.315 | .138 |

^a^energy.tm.IMF1.md_uj_accelerometer represents linear jerk feature derived from device accelerometer. Cloud coverage was determined by percentage of sky occluded by clouds, between 0 and 1, inclusive. Effect size shows the per unit increase in each weather parameter (ie, 30°F increase in temperature). All results were analyzed using a linear mixed-effects model followed by ANOVA. ANOVA, analysis of variance; DSST, Digit Symbol Substitution Test; hPa, hectopascal; MS, multiple sclerosis.
