# Supplementary material for: Evaluating the Utility of Smartphone-Based Sensor Assessments in Persons With Multiple Sclerosis in the Real-World Using an App (elevateMS): Observational, Prospective Pilot Digital Health Study
Source: JMIR Mhealth Uhealth. 2020 Oct 27;8(10):e22108. doi: 10.2196/22108 (PMC7655470; doi:10.2196/22108)
Supplement: Multimedia Appendix 14 [file mhealth_v8i10e22108_app14.docx]

**Multimedia Appendix 14.** Association between local weather conditions and PROs in participants with MS (sensitivity analysis).

|  | **Daily health vs weather** | | | | | | | | | | | | |
| --- | --- | --- | --- | --- | --- | --- | --- | --- | --- | --- | --- | --- | --- |
|  | **Atmospheric pressure**  **(hPa)** | | **Cloud coverage**  **(%)** | | **Current temperature (°F)** | | **Relative humidity**  **(%)** | | **Maximum temperature (°F)** | | **Minimum temperature (°F)** | | |
| **Health** | **Effect Size** | ***P* value** | **Effect Size** | ***P* value** | **Effect Size** | ***P* value** | **Effect Size** | ***P* value** | **Effect Size** | ***P* value** | **Effect Size** | ***P* value** |  |
| Amazing | 1017.98 | <.001 | 39.85 | <.001 | 57.21 | <.001 | 63.32 | <.001 | 60.12 | <.001 | 53.99 | <.001 |  |
| Okay | 0.36 | .460 | 4.78 | .054 | 4.79 | <.001 | 4.37 | .002 | 4.80 | <.001 | 4.86 | <.001 |  |
| So-so | 0.35 | .535 | 2.57 | .384 | 3.11 | .018 | 2.36 | .168 | 3.22 | .015 | 3.14 | .016 |  |
| Not great | 0.70 | .390 | ‒2.66 | .511 | 2.22 | .209 | 4.04 | .081 | 2.34 | .190 | 2.43 | .166 |  |

|  | **Daily mobility vs weather** | | | | | | | | | | | | |
| --- | --- | --- | --- | --- | --- | --- | --- | --- | --- | --- | --- | --- | --- |
|  | **Atmospheric pressure**  **(hPa)** | | **Cloud coverage**  **(%)** | | **Current temperature (°F)** | | **Relative humidity**  **(%)** | | **Maximum temperature (°F)** | | **Minimum temperature (°F)** | | |
| **Mobility** | **Effect Size** | ***P* value** | **Effect Size** | ***P* value** | **Effect Size** | ***P* value** | **Effect Size** | ***P* value** | **Effect Size** | ***P* value** | **Effect Size** | ***P* value** |  |
| Excellent | 1018.39 | <.001 | 41.50 | <.001 | 61.38 | <.001 | 64.47 | <.001 | 64.27 | <.001 | 58.21 | <.001 |  |
| Very good | 0.03 | .961 | 2.33 | .420 | ‒3.20 | .018 | 4.08 | .016 | ‒3.27 | .017 | ‒3.02 | .026 |  |
| Good | ‒0.03 | .961 | 3.44 | .261 | ‒2.15 | .170 | 3.65 | .050 | ‒2.15 | .174 | ‒2.05 | .188 |  |
| Not great | ‒0.71 | .277 | ‒3.75 | .294 | 2.28 | .207 | ‒1.49 | .492 | 2.73 | .134 | 1.86 | .300 |  |

|  | **Daily pain vs weather** | | | | | | | | | | | | |
| --- | --- | --- | --- | --- | --- | --- | --- | --- | --- | --- | --- | --- | --- |
|  | **Atmospheric pressure**  **(hPa)** | | **Cloud coverage**  **(%)** | | **Current temperature (°F)** | | **Relative Humidity**  **(%)** | | **Maximum temperature (°F)** | | **Minimum temperature (°F)** | | |
| **Pain** | **Effect size** | ***P* value** | **Effect size** | ***P* value** | **Effect size** | ***P* value** | **Effect size** | ***P* value** | **Effect size** | ***P* value** | **Effect size** | ***P* value** |  |
| None | 1018.09 | <.001 | 43.47 | <.001 | 59.86 | <.001 | 64.82 | <.001 | 62.75 | <.001 | 56.79 | <.001 |  |
| Mild | 0.29 | .512 | ‒1.43 | .516 | 0.56 | .548 | 2.71 | .030 | 0.73 | .439 | 0.42 | .654 |  |
| Moderate | 0.63 | .245 | 1.09 | .689 | 0.34 | .783 | 2.81 | .075 | 0.41 | .739 | 0.30 | .806 |  |
| Severe | ‒0.40 | .639 | ‒7.65 | .068 | 2.94 | .129 | ‒0.44 | .856 | 2.84 | .147 | 2.61 | .177 |  |

Cloud coverage was determined by percentage of sky occluded by clouds, between 0 and 1, inclusive. Effect size shows the correlation between each PRO metric and weather parameter and was calculated using the Amazing daily health score, Excellent daily mobility score, or None daily pain score as baseline. All results were analyzed using a linear mixed-effects model followed by ANOVA. Sensitivity analyses were conducted to evaluate the impact of extreme health, pain, and mobility categories (Horrible) on the association between PROs and local weather conditions. ANOVA, analysis of variance; hPa, hectopascal; MS, multiple sclerosis; PROs, patient-reported outcomes.
